# Surrogate modeling of electrospun PVA/PLA nanofibers using artificial neural network for biomedical applications

**DOI:** 10.1038/s41598-025-94608-8

**Published:** 2025-04-15

**Authors:** Reyhaneh Fatahian, Rasool Erfani

**Affiliations:** 1 Department of Textile Engineering, Yazd Branch, Islamic Azad University, Yazd, Iran; 2 Department of Engineering, Manchester Metropolitan University, Manchester, M1 5GD UK; 3 Department of Civil, Environmental and Geomatic Engineering, University College London, London, WC1E 6BT UK

**Keywords:** Surrogate modeling, Hydrophilicity, Wound healing, Artificial neural network (ANN), Healthcare, Biotechnology, Health care, Medical research, Engineering, Nanoscience and technology

## Abstract

Blending poly (lactic acid) (PLA) with poly (vinyl alcohol) (PVA) improves the strength and hydrophilicity of nanofibers, making them suitable for biomedical applications like wound dressings. This study explores how electrospinning parameters—applied voltage, flow rate, and needle-to-collector distance—affect PVA/PLA nanofiber properties, optimizing them using a Taguchi design of experiment (DoE) approach to enhance their mechanical and surface properties for clinical use. Given the high costs and time associated with conducting extensive experimental tests, an artificial neural network based surrogate model is developed to predict experimental outcomes more efficiently, facilitating faster identification of optimal design configurations. Analysis of Variance reveals flow rate as the most significant determinant of fiber diameter. The optimal electrospinning configuration yields nanofibers with an average diameter of 127.6 ± 19.8 nm. These fibers exhibit exceptional tensile strength, flexibility, and a water contact angle of 37°, demonstrating superior hydrophilicity conducive to cell adhesion and proliferation—key factors in promoting wound healing. Comparative analyses confirm that the optimized scaffold (18 cm needle-to-collector distance, 0.6 ml/h flow rate, and 18 kV applied voltage) significantly outperforms alternative configurations, such as 10 cm needle-to-collector distance, 1.2 ml/h flow rate, and 22 kV applied voltage, which display larger diameters, reduced hydrophilicity (contact angle of 72°), and diminished suitability for medical use. Validation experiments affirm the accuracy and reproducibility of the Taguchi optimization, substantiating the methodological rigor and reliability of the findings. This work contributes novel insights into the tunable design of electrospun nanofibers, providing a pathway to developing advanced wound dressings that facilitate tissue integration and accelerate healing. The optimized PVA/PLA nanofibers have the potential to revolutionize wound care by offering a cost-effective and clinically viable solution for enhancing patient recovery, reducing treatment durations, and improving global healthcare outcomes.

## Introduction

Recent years have seen growing interest in one-dimensional nanomaterials, such as nanofibers, nanobelts, nanowires, and nanorods, due to their unique properties in optics, thermal dynamics, electrical conductivity, and mechanics. These materials have diverse applications in fields like biomedical engineering, catalysis, energy storage, and gas sensing^1–3^. Nanotechnology, particularly electrospinning, has emerged as a promising tool in tissue engineering and regenerative medicine^4,5^. Electrospinning is an efficient method for producing nanofibrous structures by using an electric field to draw fine fibers from polymer solutions, creating meshes that mimic the natural extracellular matrix. This technique allows precise control over key properties like fiber diameter, porosity, and surface area, improving the performance of wound dressings. The resulting nanofibers, with their high surface-to-volume ratios, promote gas exchange, moisture regulation, offer a protective barrier and mechanical properties. They also enable the controlled release of therapeutic agents, accelerating the healing process^6–10^. Electrospun wound dressings provide superior strength, flexibility, and porosity, facilitating cell infiltration and tissue regeneration, making them a promising solution for advanced wound care in healthcare and regenerative medicine^11,12^.

The development of advanced fiber materials for applications in textiles, composites, and biomedical devices has focused on improving mechanical and structural properties. Polyvinyl alcohol (PVA) and poly (lactic acid) (PLA) are two key polymers known for their biocompatibility, biodegradability, and versatility. Recent research on hybrid PVA/PLA fibers has shown promise, combining the strengths of both materials^6^. Polylactic acid (PLA) is a widely used biopolymer known for its excellent biodegradability and biocompatibility, making it ideal for biomedical applications such as drug delivery, gene transfer, surgical sutures, and tissue engineering^13,14^. PLA’s properties enhance its suitability for these uses, often in combination with other biocompatible polymers like poly (glycolic acid) (PGA), Polyvinyl alcohol (PVA) and poly(ε-caprolactone) (PCL) in electrospinning^15^. This versatility extends to PLA nanofibers, particularly in regenerative medicine. Studies have demonstrated the potential of PLA/curcumin nanofibers in regenerative medicine, highlighting their exceptional blood compatibility and wound healing properties^16,17^. Also, PLA fibers alone, while exhibiting excellent tensile strength and rigidity, often suffer from brittleness and low elongation at break, limiting their applicability in flexible materials^18,19^.

Polyvinyl alcohol (PVA) is a water-soluble, non-toxic, biocompatible, and biodegradable polymer widely studied for biomedical and textile applications, especially for its compatibility with water-based solvents, making PVA electrospinning a popular research area^20–22^. Sawada et al.^23^ used a water-DMF blend to enhance PVA electrospinning, noting that DMF’s low surface tension, low vapor pressure, and higher boiling point improved the process. PVA nanofibers, on the other hand, are more flexible but exhibit lower tensile strength and are prone to water absorption, which can weaken their mechanical properties over time. By blending these two polymers, PVA/PLA fibers are hypothesized to exhibit superior toughness and flexibility while maintaining a high level of strength^24^. Unlike other fiber blends that may suffer from phase separation or reduced mechanical performance, PVA/PLA fibers can be tailored to achieve an optimal balance of properties. By modifying the PVA/PLA ratio, their performance can be precisely adjusted, making them well-suited for next-generation biomedical applications. Kaniuk et al.^25^ in their study explores electrospun PLA/PVA scaffolds for skin regeneration, investigating sequential and concurrent systems with different PLA: PVA ratios. Removing PVA fibers increased porosity from 78 to 99% and reduced water absorption time. Chemical analysis confirmed PVA residue, while in vitro studies showed improved cell penetration, enhancing scaffold permeability for cells and nutrients. In another study, Zhong et al.^26^ developed RA-BSP-PVA@PLA nanofibers for wound healing using coaxial electrospinning. The fibers, characterized by SEM, CLSM, and FTIR, displayed a core–shell structure, air permeability, flexibility, and biocompatibility. In rat models, they enhanced tissue growth, macrophage proliferation, and wound healing, demonstrating strong potential as wound dressings.

Electrospun nanofibers are highly effective in wound healing due to their ability to support cell migration and proliferation, promoting tissue formation^5^. In open wounds, they aid in hemostasis, fluid absorption, gas exchange, and cell respiration. Wound healing scaffolds must also prevent contamination and infections while accelerating healing^27,28^. Biopolymer-based nanofibers must offer biocompatibility, biodegradability, antibacterial properties, and controlled drug delivery^5^. Electrospinning is a rapidly advancing technique for creating 2D and 3D nanofiber structures, allowing precise control over morphology, including size and diameter^29–33^. Numerous studies have investigated the parameters of the electrospinning process, highlighting their significant influence on nanofiber shape and diameter. Lee et al.^34^ studied the effects of solvent systems on PCL nanofibers and found that methylene chloride (MC) was the only solvent that dissolved the polymer, though it caused frequent interruptions due to its low boiling point. Adding dimethylformamide (DMF) improved the electrospinning process, producing finer nanofibers by reducing viscosity and increasing electrical conductivity. For PLA nanofibers, research has focused on polymer concentration and process parameters, such as electric field strength, feed rate, and needle-to-collector distance, which significantly affect nanofiber morphology. Gu and Ren^35^ dissolved amorphous PLA (PDLA) in a chloroform-acetone mixture (2:1 v/v) and used a design of experiments (DOE) approach to optimize and predict nanofiber morphology and diameter. They found that polymer concentration significantly influenced nanofiber diameter, with higher concentrations and applied voltages resulting in more uniform fibers. Several studies have explored the modification of PLA’s properties to enhance its functionality. For instance, Wang et al.^36^ blended PLA with poly(γ-Benzyl-L-glutamate) (PBLG), improving its surface wettability. Maleki et al.^37^ produced PVA-PLA nanofibers by incorporating AgNO₃ nanoparticles via coaxial electrospinning. Their results revealed that the nanofiber mats exhibited enhanced antibacterial activity against *Escherichia coli* (Gram-negative) and *Staphylococcus aureus* (Gram-positive), with inhibition zones ranging from 1 to 2 mm correlating to increased silver content. Bi et al.^38^ used electrospinning to create highly porous PLA and PLA/PVA/SA nanofiber membranes for wound healing. Achieving optimal conditions is challenging due to the interdependence of various parameters. The Taguchi method offers a robust optimization strategy, using an orthogonal array (OA) to manage experimental factors and a signal-to-noise ratio (S/N) to identify reliable conditions despite result variations^39,40^.

In electrospun nanofiber production for wound dressings, parameters like polymer concentration, applied voltage, flow rate, and needle-to-collector distance are crucial in determining fiber properties. The Taguchi method systematically analyzes these factors, optimizing key characteristics such as fiber diameter, porosity, and mechanical strength. This approach improves reproducibility and ensures consistent performance in biomedical applications, advancing materials science for effective wound care solutions^40–43^. Nanofibers, with their high surface-to-volume ratio and versatile polymer compositions, effectively deliver therapeutic agents and absorbents. Optimized nanofiber dressings create ideal wound-healing environments, highlighting the importance of methods like Taguchi for superior outcomes. While polylactic acid (PLA) is biocompatible and biodegradable, its brittleness and hydrophobicity can be addressed by blending it with hydrophilic, elastic polymers like polyvinyl alcohol (PVA), improving elasticity and hydrophilicity. In this study, the PVA/PLA blend is employed to produce nanofibers with desirable properties, aiming to address existing challenges. Notably, while the structure and diameter distribution of electrospun nanofibers have been extensively studied, the specific influence of process parameters on single-nozzle electrospinning of PVA/PLA nanofibers using a mutual solvent remains unexplored in the literature. This research is dedicated to evaluating the feasibility of producing PVA/PLA nanofibers through electrospinning, providing insights into optimizing process parameters for improved performance.

With the rapid advancements in artificial intelligence (AI) and machine learning, the use of advanced data-driven algorithms for predictive modeling has become increasingly significant. Among these, artificial neural networks (ANNs) have proven highly effective in capturing complex, nonlinear relationships between design parameters and performance metrics. Their versatility has enabled their widespread application in diverse fields, including fluid dynamics^44^, biomedical engineering^45^, and textile fabrication^46^, establishing them as powerful tools for optimizing performance in various domains.

The existing literature on electrospun nanofibers primarily focuses on single-variable optimization approaches, which fail to capture the intricate, nonlinear interactions among multiple processing parameters. Traditional empirical and physics-based models often struggle to accurately predict fiber diameter and morphology due to the complexity of the electrospinning process. Additionally, conventional experimental designs, such as full-factorial approaches, require a large number of trials, making the optimization process resource-intensive and inefficient. While artificial neural networks (ANNs) have been successfully applied in various engineering fields, their potential as surrogate models for electrospinning optimization remains largely underexplored. Furthermore, many studies focus on general fiber formation without specifically tailoring nanofiber properties for biomedical applications, where precise control over diameter and morphology is crucial for biocompatibility and functionality.

This study hypothesizes that an ANN-based surrogate modeling approach can effectively capture the nonlinear relationships between key electrospinning parameters and fiber diameter, overcoming the limitations of traditional empirical and physics-based models. By integrating an ANN with a Design of Experiments (DoE) strategy using the Taguchi method, the study aims to develop an efficient and accurate predictive framework for optimizing nanofiber properties. The ANN model is expected to generalize across different electrospinning conditions, enabling precise control over fiber morphology while minimizing the experimental burden.

The novelty of this research lies in the integration of ANN as a surrogate model for electrospinning optimization, a methodology not widely explored in previous studies. Unlike conventional approaches, this study employs a multi-variable optimization strategy, considering three key parameters—needle tip-to-collector distance, flow rate, and applied voltage—simultaneously to achieve optimal nanofiber formation. The use of an L9 orthogonal array within the Taguchi methodology significantly reduces the number of required experiments while ensuring robust optimization. Moreover, the study specifically tailors the properties of PVA/PLA nanofibers for biomedical applications, addressing a critical gap in the literature by optimizing fiber morphology to enhance biocompatibility and functional performance. By leveraging machine learning techniques, this research not only improves predictive accuracy but also establishes a scalable and resource-efficient framework for nanofiber optimization.

## Methodology

### Electrospinning procedure

**Fig. 1:**
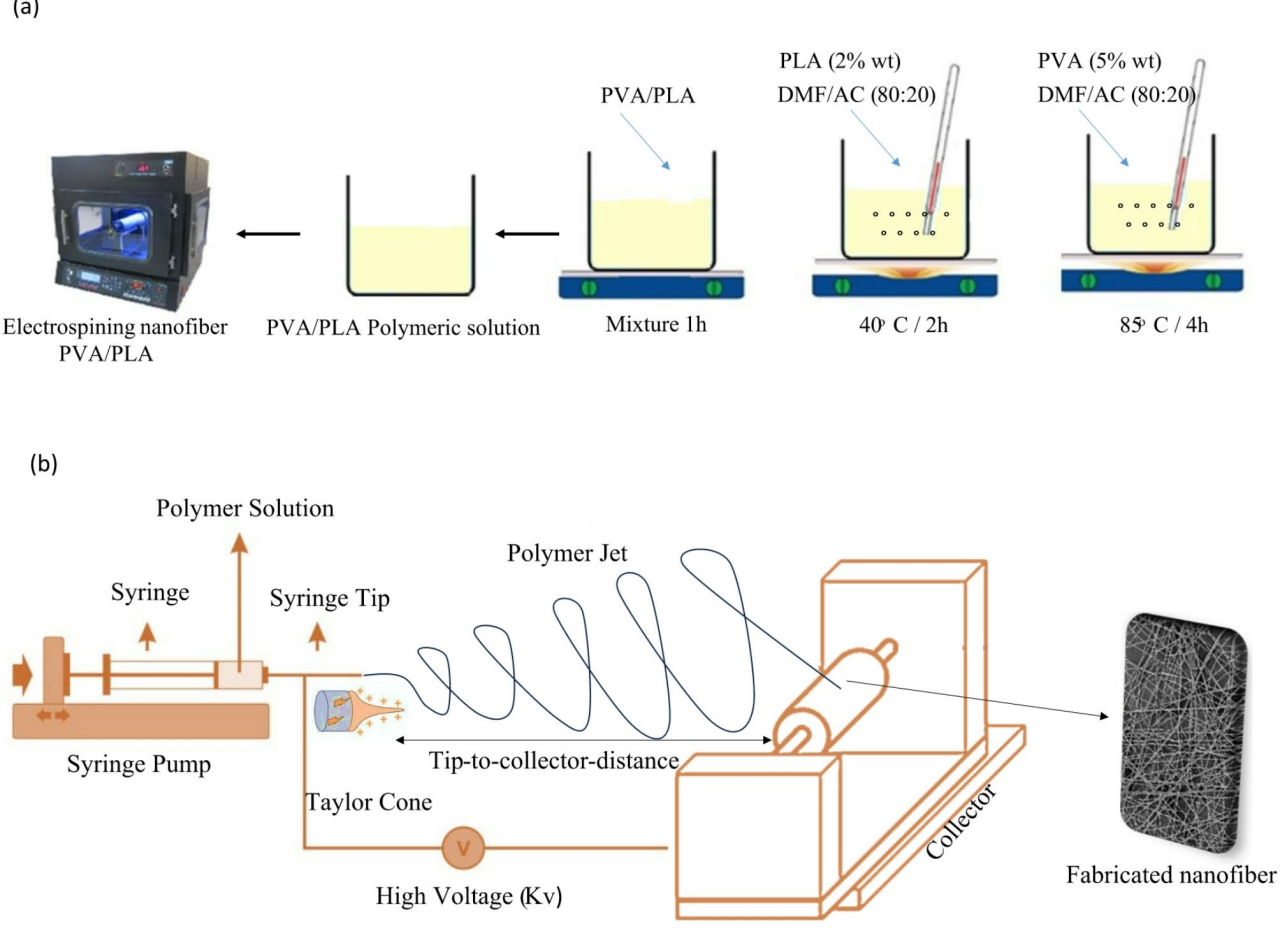
(**a**) Schematic diagram illustrating the electrospinning process, highlighting key steps involved. (**b**) Diagram of the electrospinning machine setup, showing the arrangement of components for fiber production.

In the current study, the following procedures are employed to formulate a PVA/PLA solution. First, a 5 wt% PVA solution is prepared by dissolving PVA (Mw = 89,000, obtained from Sigma-Aldrich, USA) in a mixture of N, N-Dimethylformamide (DMF, Sigma-Aldrich) and Acetone (AC, Sigma-Aldrich) at an 80:20 ratio. This dissolution process is carried out for 4 h at 85 °C. Similarly, a 2 wt% PLA solution (Mw = 60,000, obtained from Sigma-Aldrich, USA) is prepared by dissolving PLA in the same DMF/AC mixture at the same solvent ratio, for 2 h at 40 °C. After preparing both solutions, the PVA and PLA solutions are combined in a 50:50 (v/v) ratio to form the spinning solution. The PVA/PLA mixture is then stirred on a magnetic stirrer for 1 h to ensure homogeneity. Figure 1 provides a visual representation of the procedural steps, offering a clear overview of the experimental setup and methodology employed in this study.

The electrospinning setup comprises a syringe pump, a high-voltage power supply, and a collector plate covered with non-stick aluminum foil for easy mat removal. The solutions are loaded into 5 ml syringes, which are positioned horizontally on the syringe pump. The pump delivers the solution to the tip of an 18G metal needle (Terumo, Belgium), where a voltage is applied. The nanofibers are collected onto the aluminum foil. The electrospinning chamber is enclosed to maintain a consistent environment, ensuring stable temperature and humidity levels throughout the process. This controlled environment is critical for achieving optimal electrospinning conditions, with the procedure conducted at a temperature of 30 °C and a relative humidity of approximately 25%. Maintaining such stability is vital for producing high-quality fibers, as it minimizes fluctuations that could negatively impact the electrospinning process and the properties of the resulting nanofibers.

During the electrospinning process, the solvent rapidly evaporates as the polymer solution is exposed to the electric field. However, some residual solvent, particularly DMF, may remain in the nanofibers after spinning. To ensure the complete removal of any residual solvents, an additional drying step is typically necessary. In this study, the electrospun mat was placed under a fume hood to facilitate the evaporation of these residual solvents.

### Morphological characterization

The surface morphology of the nanofibers is examined using a Field-Emission Scanning Electron Microscope (FE-SEM), specifically the Sigma model from Zeiss (Germany). This advanced microscopy technique provides exceptional resolution, allowing for a detailed inspection of nanofiber structures at the nanoscale. The FE-SEM enables the visualization of key surface features, such as roughness, porosity, and orientation, which are essential for understanding the nanofibers’ behavior and properties in various applications.

To quantitatively analyze the structural characteristics of the composite nanofibers, ImageJ software—a widely used tool for image processing—is employed. ImageJ allows for precise measurements of different sections of individual nanofibers captured in the SEM images, facilitating the determination of their mean diameter and diameter distribution. This quantitative analysis is crucial for evaluating the uniformity, size distribution, and overall quality of the composite nanofibers. To calibrate the pixel-to-length ratio in the SEM images, the scale bar length is referenced. Following this calibration, the length of each nanofiber perpendicular to its axis is manually measured. This process is repeated for every fiber in the image, with approximately 40 measurements typically taken per SEM image. The average nanofiber diameter and its standard deviation are then calculated based on these measurements, providing a comprehensive assessment of the fiber properties.

### Measurement of mechanical properties

The mechanical properties of the electrospun nanofibers are evaluated using a tensile strength testing device, model DY30/31, manufactured in France. For the test, the samples are initially cut into dimensions of 10 × 50 mm. The ends of each specimen are then placed in the upper and lower grips of the device, with the distance between the jaws set to 30 mm. The jaws are separated at a rate of 10 mm/min until the samples broke. During the testing process, the maximum elongation and tensile strength of the specimens are recorded^22^.

### Contact angle

The water contact angle measurement is employed to evaluate the hydrophilicity of the PVA/PLA scaffold in a 50:50 (v/v) ratio under specific electrospinning conditions, including feeding rates of 0.6 and 1.2 ml/h, a voltage of 22 kV, and needle-to-collector distances of 10 and 18 cm. To measure the contact angle, a water droplet is placed on the surface of each nanofiber. The droplet is captured using a Canon EOS 400D camera with an 18–55 mm lens under controlled environmental conditions. The contact angle is then measured from the captured image, which displayed a square geometry^27^.

### ANN-based surrogate model

#### Data collection using design of experiment (DoE)

The Response Surface Methodology (RSM) and the Taguchi technique^47,48^ are widely used methodologies in the implementation of DoE. The Taguchi method, known for its reliability and cost-effectiveness, helps identify the optimal system design with minimal experimentation. It is particularly valuable in various problem-solving contexts where optimizing parameters and processes is essential to achieving quality, efficiency, and cost-effectiveness. The robustness and efficiency of the Taguchi method make it a powerful tool in these applications^49–51^.

**Fig. 2:**
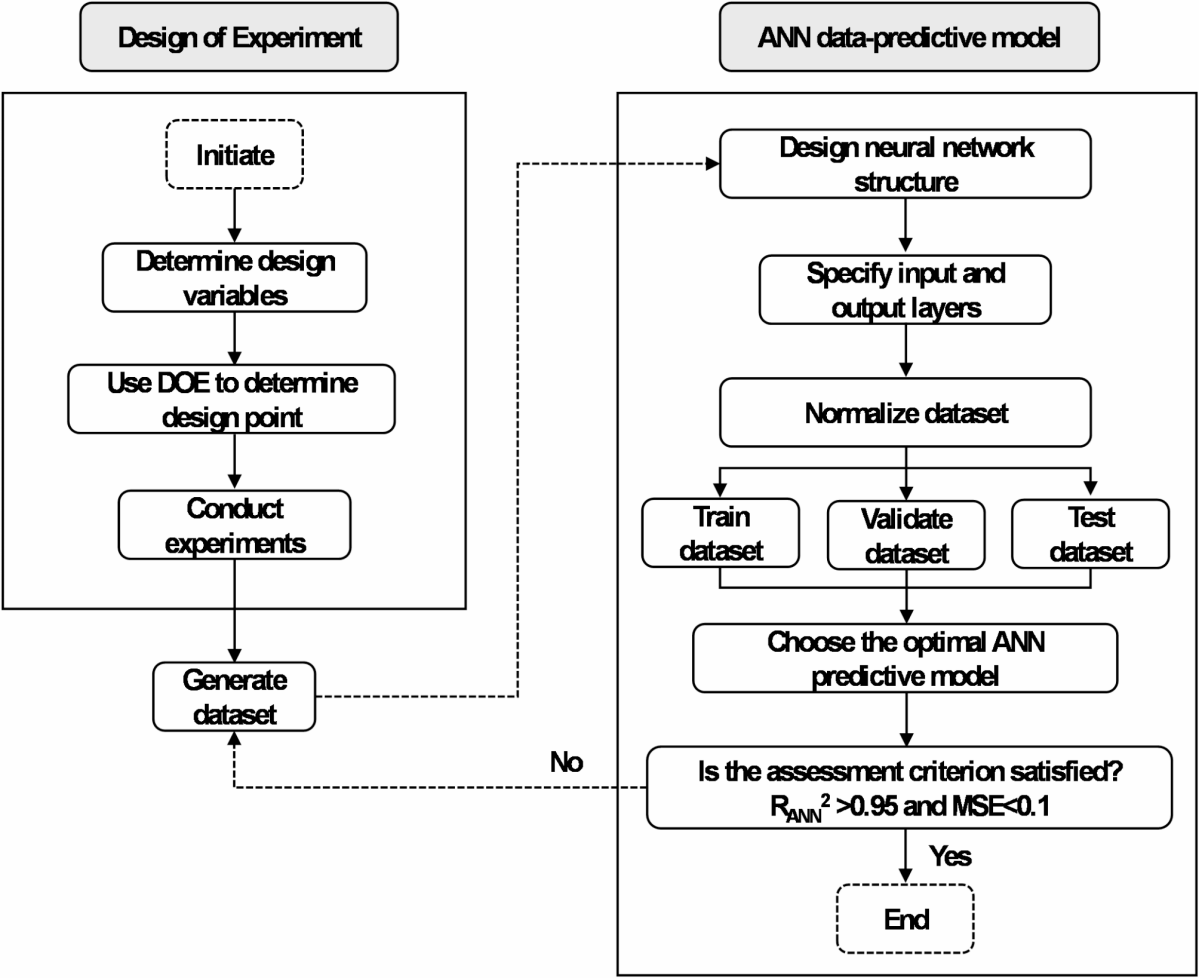
Flowchart outlining the complete optimization framework.

This section outlines the optimization approach employed in the current study, focusing on the DoE process. The Taguchi DoE method is used to determine the optimal combination of parameters—such as tip-to-collector distance, flow rate, and voltage—to minimize both the diameter size and its variation in electrospun polyvinyl alcohol/polylactic acid (PVA/PLA) nanofibers. The principal objective function of this investigation is to reduce the nanofiber diameter. The optimization approach is illustrated in Fig. 2, showing the process of selecting design parameters, choosing an orthogonal array (OA) for the design variables, conducting experiments based on the OA, and evaluating the results within the Taguchi framework.

In the Taguchi technique, the Signal-to-Noise (S/N) ratio is a statistical measure of performance, aiming to identify the optimal parametric combinations. In this context, “noise” refers to undesirable variations, while “signal” represents the desired or predicted values. The average S/N ratio reflects the distinct effect of each factor level on the response, calculated by averaging the S/N ratios across the experimental cases where each factor level is applied.

Three categories of S/N ratios are defined based on the nature of the predicted system responses: larger-the-better (LB), nominal-the-better (NB), and smaller-the-better (SB). Since the objective of this study is to minimize both the nanofiber diameter and its variance, the Taguchi approach indicates that the highest S/N ratio corresponds to the optimal condition. Therefore, the optimum design parameters are determined using the smaller-the-better (SB) S/N ratio principle, as defined below:

1 $$S/N = - 10\log \left( {\sum\limits_{i = 1}^{n} {\frac{1}{n}y_{i}^{2} } } \right)$$

n is the number of experiments and y_i_ denotes nanofiber diameter.

Choosing factors and levels that maximize the Signal-to-Noise (S/N) ratio is essential for determining the optimal value, as this ratio reflects the impact of noise on the system response. In the present study, three key factors—needle tip-to-collector distance, flow rate, and voltage—are selected, each with three levels. These values are specifically chosen to achieve thin, smooth fibers without beads. Outside these ranges, no desirable nanofibers were observed on the collector. After establishing the design parameters and their respective levels, experiments are organized using orthogonal arrays. The L_9_ orthogonal array is employed, allowing for the execution of nine distinct experiments. Table 1 provides a detailed summary of the factors and their levels in relation to the control parameters.

**Table 1:** Relevant data regarding variables and levels that are associated with control parameters.

Factors	Control parameters	Units	Notations	Levels		
				1	2	3
A	Needle tip-to-collector distances	cm	D	10	14	18
B	Flow rates	ml/h	F	0.6	0.9	1.2
C	Voltages	kV	V	18	20	22

Performing a full factorial Design of Experiments (DoE) require 3^3^ = 27, which can be both cost-prohibitive and time-consuming. To avoid exhaustively testing all possible combinations, the Taguchi method offers a more economical alternative by using an orthogonal array (OA) to explore parameter dependencies with a reduced number of tests. In this study, the standard OA L_9_ (3^3^) orthogonal array is employed, which allows for the evaluation of three factors, each at three levels, as outlined in Table 2. This approach enables efficient investigation of the key factors’ influence while minimizing the number of required experiments.

**Table 2:** L_9_ (3^3^) orthogonal array.

Experiment	Factors (Coded)			Factors (Uncoded)		
	A	B	C	Needle tip-to-collector distances	Flow rates	Voltages
E1	1	1	1	10	0.6	18
E2	1	2	2	10	0.9	20
E3	1	3	3	10	1.2	22
E4	2	1	2	14	0.6	20
E5	2	2	3	14	0.9	22
E6	2	3	1	14	1.2	18
E7	3	1	3	18	0.6	22
E8	3	2	1	18	0.9	18
E9	3	3	2	18	1.2	20

#### Artificial neural network (ANN) structure

**Fig. 3:**
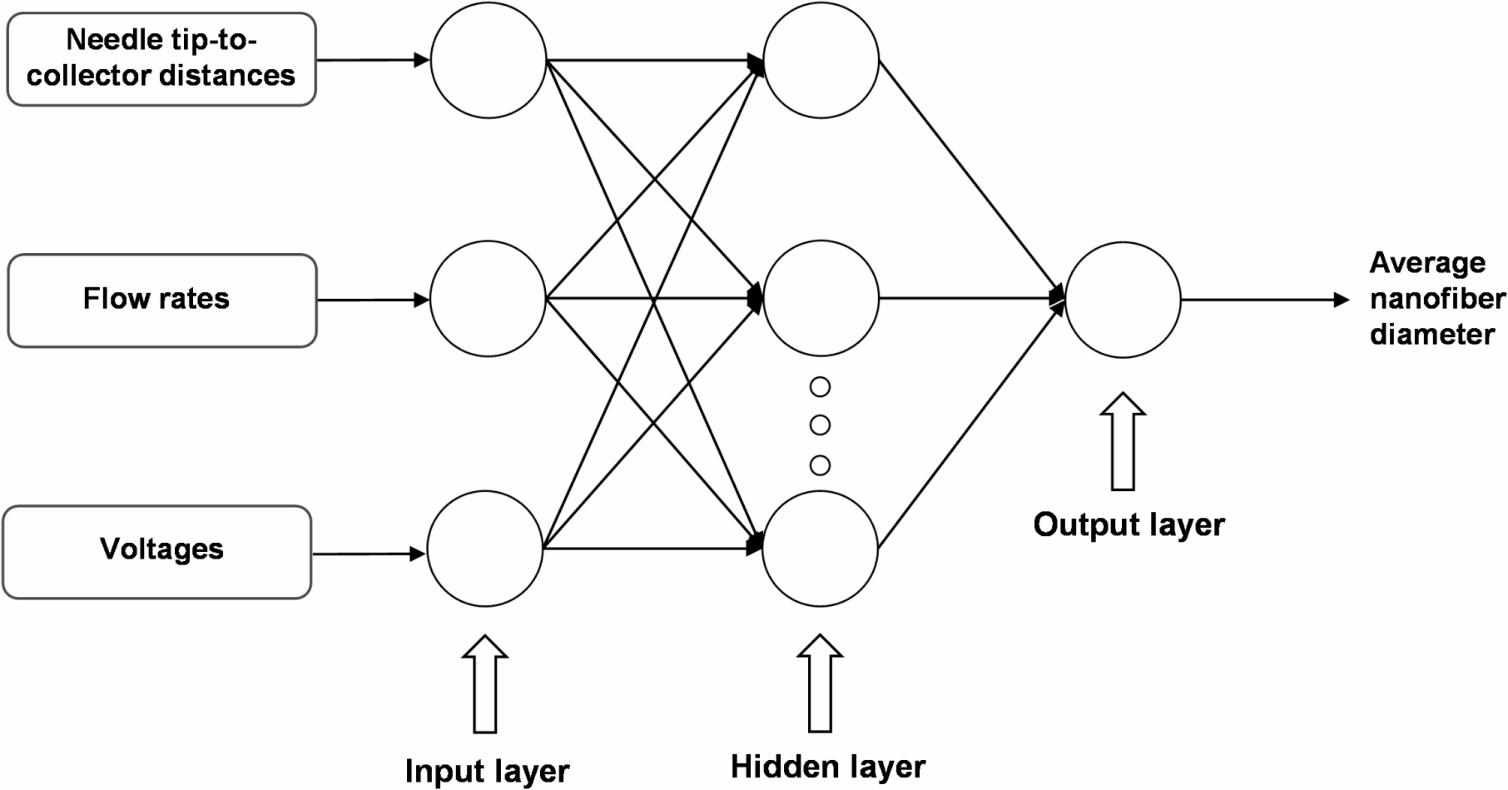
Artificial neural network (ANN) theoretical structure.

The ANN architecture consists of three-layers: an input layer, a hidden layer, and an output layer. Initially configured with 10 neurons, hidden layer size is increased to 12 for achieving desired accuracy during the setup of network. The Levenberg–Marquardt (LM) learning algorithm is selected for its proven robustness, reliability, and fast convergence in predicting the response to input data. The data, obtained using the orthogonal array DoE method, are divided into three groups: 80% for training, with the remaining 20% split evenly between validation and testing. This data partitioning is critical for effectively training and evaluating the performance of machine learning models. Figure 3 outline the structure of the ANN and its training process. The model’s accuracy is assessed by using performance metrics such as Mean Squared Error (MSE) and the Coefficient of Determination (${\text{R}}_{ANN}^{2}$). These metrics are critical to quantify the model’s predictive accuracy and reliability.

## Results and discussion

The results are presented in three sections. "Nanofiber morphology and diameter" section showcases the SEM micrographs of electrospun nanofiber morphology based on the L_9_ orthogonal array (OA). In "Assessing the Taguchi and ANOVA approaches" section, the data are analysed using both the Taguchi method and ANOVA. The Taguchi method allows for the simultaneous investigation of multiple factors by evaluating the Signal-to-Noise (S/N) ratio across all experiments, while ANOVA provides additional insights into the significance of individual factors and their interactions on nanofiber diameter. Finally, "Confirmation test" section presents a confirmation test to verify the accuracy and reliability of the Taguchi method applied in optimizing the design parameters.

### Nanofiber morphology and diameter

Solvent selection plays a crucial role in preparing electrospinning solutions and ensuring a stable, reversible electrospinning process. In this study, a DMF/acetone (80:20) solvent system was chosen for dissolving PVA and PLA. The high proportion of DMF (80%) was selected due to its ability to effectively dissolve both polymers, acting as a bridge solvent that facilitates homogeneous blending and prevents phase separation, thereby promoting uniform fiber formation.

The DMF content in the solvent system significantly impacts fiber morphology, as higher DMF concentrations reduce surface tension and viscosity, leading to finer fiber diameters. By utilizing 80% DMF, the fiber diameter was precisely controlled to achieve the desired morphology. Although DMF is toxic, its superior solubility properties, stabilization of the electrospinning process, and role in ensuring uniform fiber formation made it essential for this study. To minimize residual DMF, extended air-drying with controlled ventilation was employed. Additionally, acetone served as a secondary solvent to enhance evaporation. This study primarily focuses on optimizing the electrospinning process, which will be discussed in detail in the following sections.

Upon completing all experiments based on the L_9_ orthogonal array design, the diameters of the electrospun PVA/PLA nanofibers were meticulously measured using SEM micrographs and ImageJ software. The resulting images, showcasing the intricate morphologies of the nanofibers, are presented in Fig. 3 for detailed analysis. The electrospun PVA/PLA nanofibers exhibited smooth, bead-free morphologies with uniform distribution. To further enhance the understanding of the optimization study, frequency contribution diagrams were generated, focusing on the diameter range of 127.6 ± 19.8 nm to 307.6 ± 57.5 nm. These diagrams provide valuable insights into the relative influence of various factors on achieving nanofiber diameters within this specific range. This in-depth analysis aids in understanding the underlying mechanisms and optimizing the electrospinning process to precisely control the characteristics of the nanofibers. The fabricated nanofibers demonstrated small diameters with minimal variation. Initial observations from the SEM images and distribution diagrams indicated that nanofibers produced under condition E3 had the largest diameter, while those produced under condition E7 exhibited the smallest diameter and the most consistent fiber diameter distribution. It can be concluded that higher voltage, increased distance between the needle and collector, and a lower flow rate tend to yield finer nanofiber distributions.

**Fig. 4:**
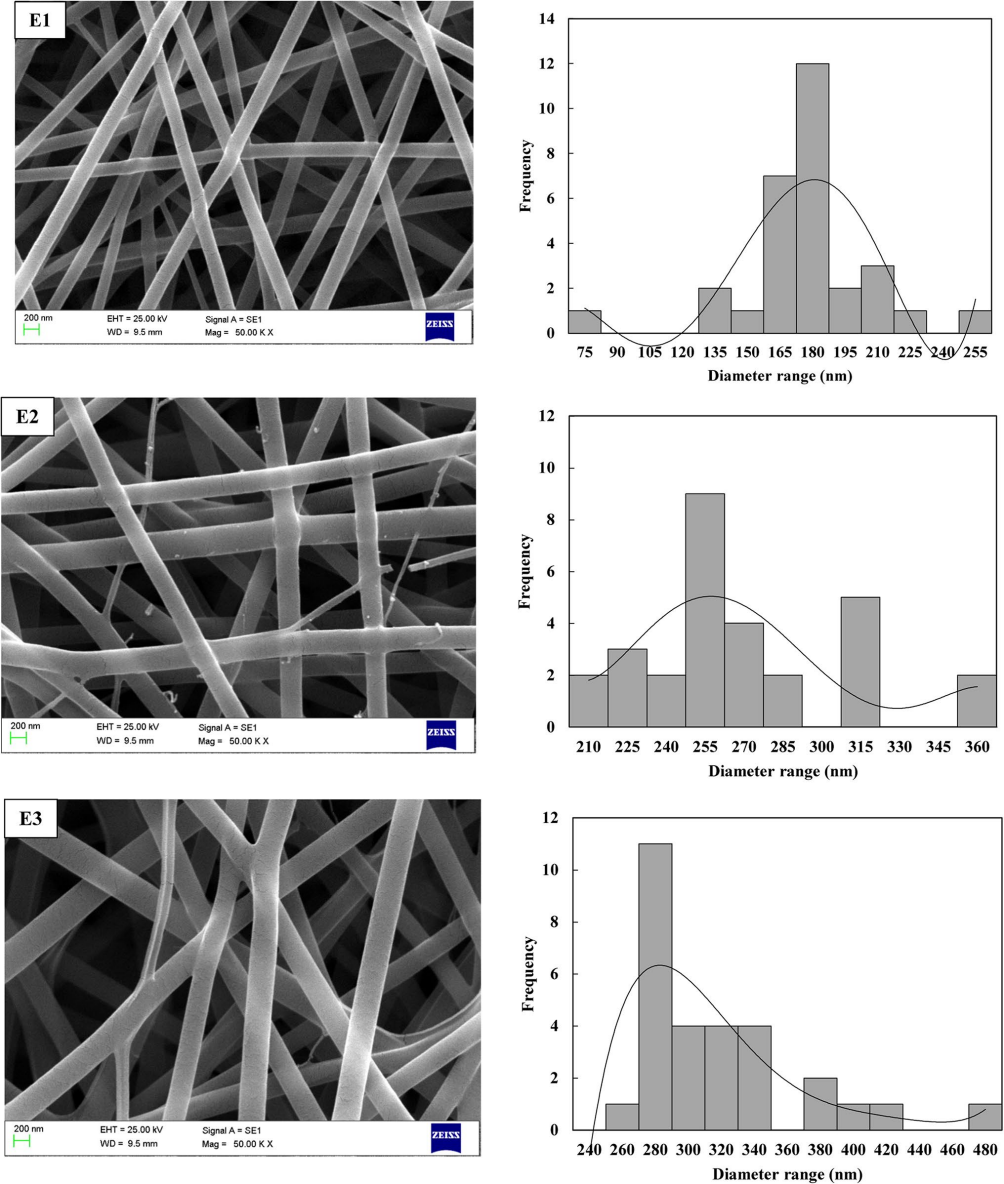

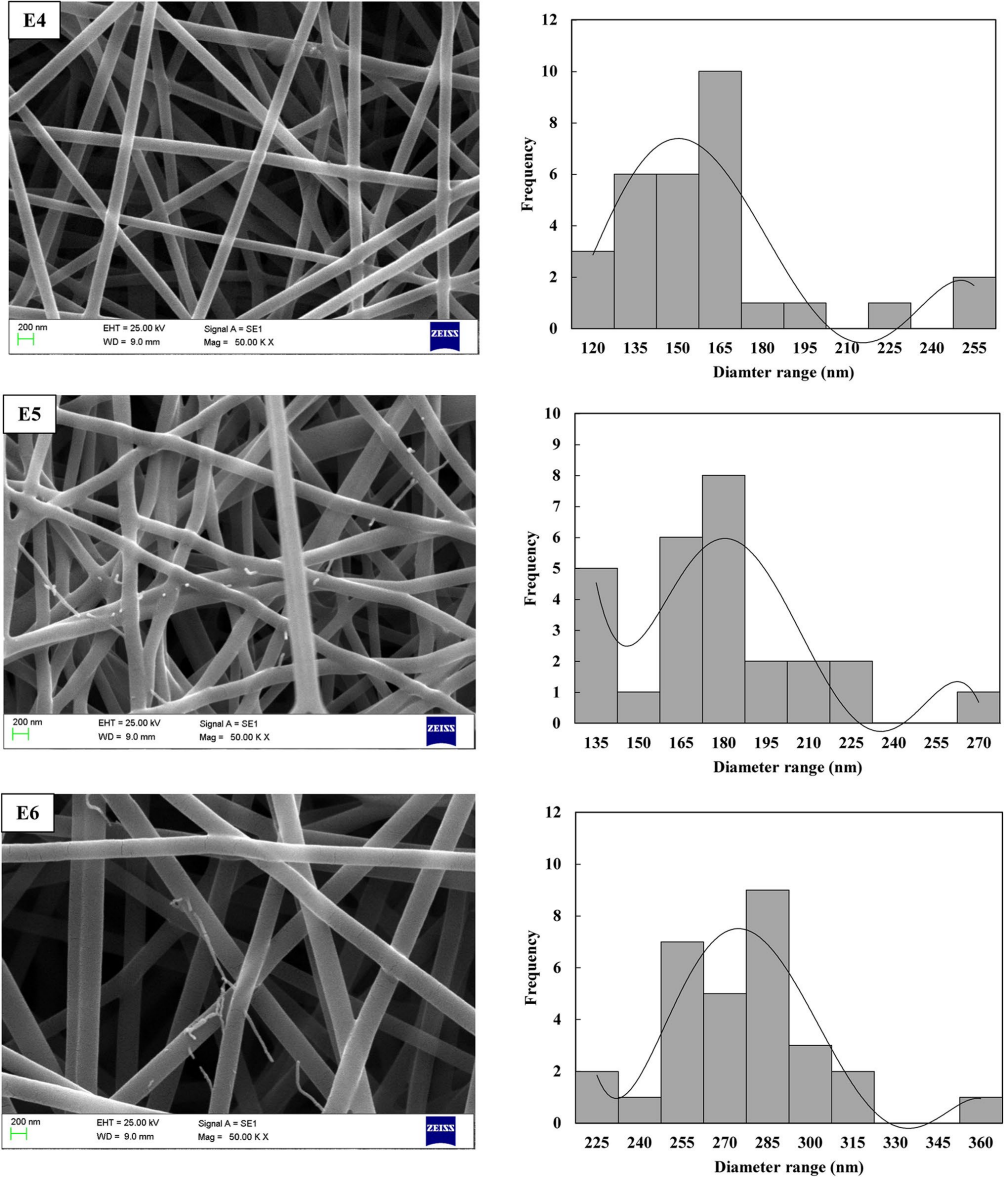

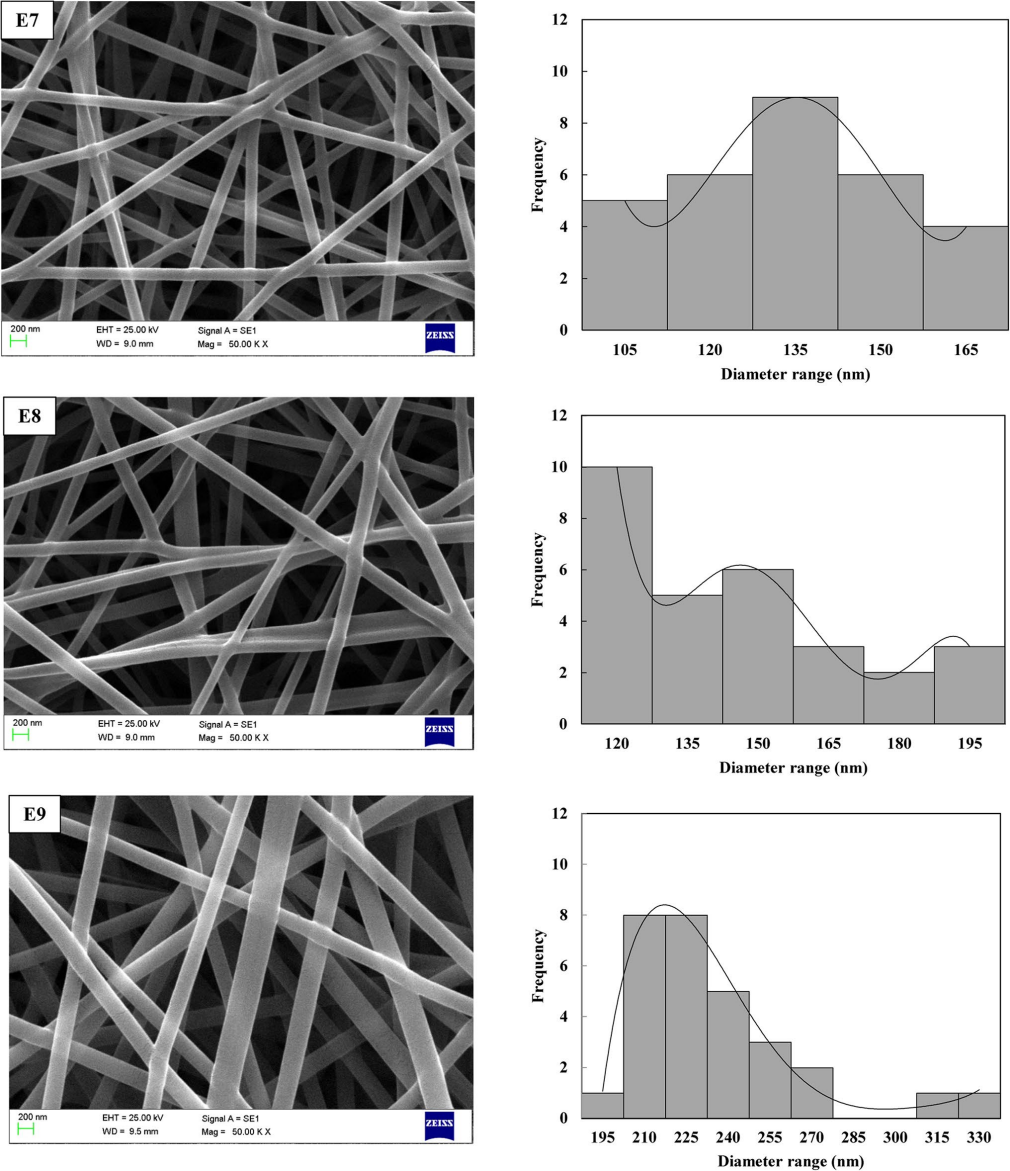
SEM images of electrospun nanofibers and their corresponding frequency distribution across diameter ranges for experiments E1–E9 using the Taguchi method.

In analyzing the impact of voltage variations on nanofiber morphology, as shown in Fig. 4 and Table 3, it was observed that increasing the electric field strength typically results in thinner fibers. However, at higher applied voltages, there was also an increase in the variability of fiber diameters. This phenomenon can be attributed to fluctuations in the electric field strength at elevated voltages. It has been noted that increasing the voltage up to a certain threshold leads to a reduction in fiber diameter, but after reaching a critical voltage, the trend reverses, causing the fiber diameter to increase. This occurs because the fiber’s flight time becomes too short at higher voltages, limiting its ability to stretch sufficiently before the solvent evaporates. Conversely, when the voltage exceeds this threshold, the diameter decreases again as the single jet splits into multiple jets. The applied voltage and the distance between the needle and collector play crucial roles in regulating the electric field strength. Increasing the applied voltage while decreasing the collector distance enhances the electric field intensity. On the other hand, the distance from the collector to the needle influences both the solvent evaporation rate and the deposition time of the polymer molecules. As the distance increases, the evaporation rate accelerates, leading to thinner, bead-free fibers, as shown in Fig. 3. Therefore, to achieve uniform, bead-free fibers, an optimal distance between the needle and collector is essential. Additionally, Fig. 3 and Table 3 illustrate the effect of the solution feeding rate, which is another key parameter in the electrospinning process. The results show that increasing the feeding rate from 0.6 to 1.2 ml/h leads to an increase in nanofiber diameter. This increase occurs because a higher feeding rate allows more polymer solution to be drawn from the needle tip, which can result in thicker fibers.

**Table 3:** Nanofiber diameters with standard deviation and their corresponding S/N ratios according to “smaller the better”.

Experiment	Combination of factors/levels	Average nanofiber diameter (nm)	S/N ratio
E1	A_10_B_0.6_C_18_	167.1 ± 33.9	− 44.460
E2	A_10_B_0.9_C_20_	255.3 ± 49.4	− 48.141
E3	A_10_B_1.2_C_22_	307.6 ± 57.5	− 49.760
E4	A_14_B_0.6_C_20_	154.0 ± 34.8	− 43.750
E5	A_14_B_0.9_C_22_	186.8 ± 62.5	− 45.428
E6	A_14_B_1.2_C_18_	269.2 ± 28.2	− 48.602
E7	A_18_B_0.6_C_22_	127.6 ± 19.8	− 42.117
E8	A_18_B_0.9_C_18_	143.3 ± 38.7	− 43.125
E9	A_18_B_1.2_C_20_	231.6 ± 41.6	− 47.295

### Assessing the Taguchi and ANOVA approaches

The average diameter of nanofibers and their corresponding S/N ratios were determined from nine experiments arranged according to the orthogonal array, as shown in Table 3. It was found that higher S/N ratios correspond to smaller nanofiber diameters. Among the experimental cases, E7 emerged as the optimal condition, demonstrating an average diameter of 127.6 ± 19.8 nm and an S/N ratio of − 42.1. In contrast, E3 yielded the least favorable result, with an average diameter of 307.6 ± 57.5 nm and an S/N ratio of − 49.8.

**Fig. 5:**
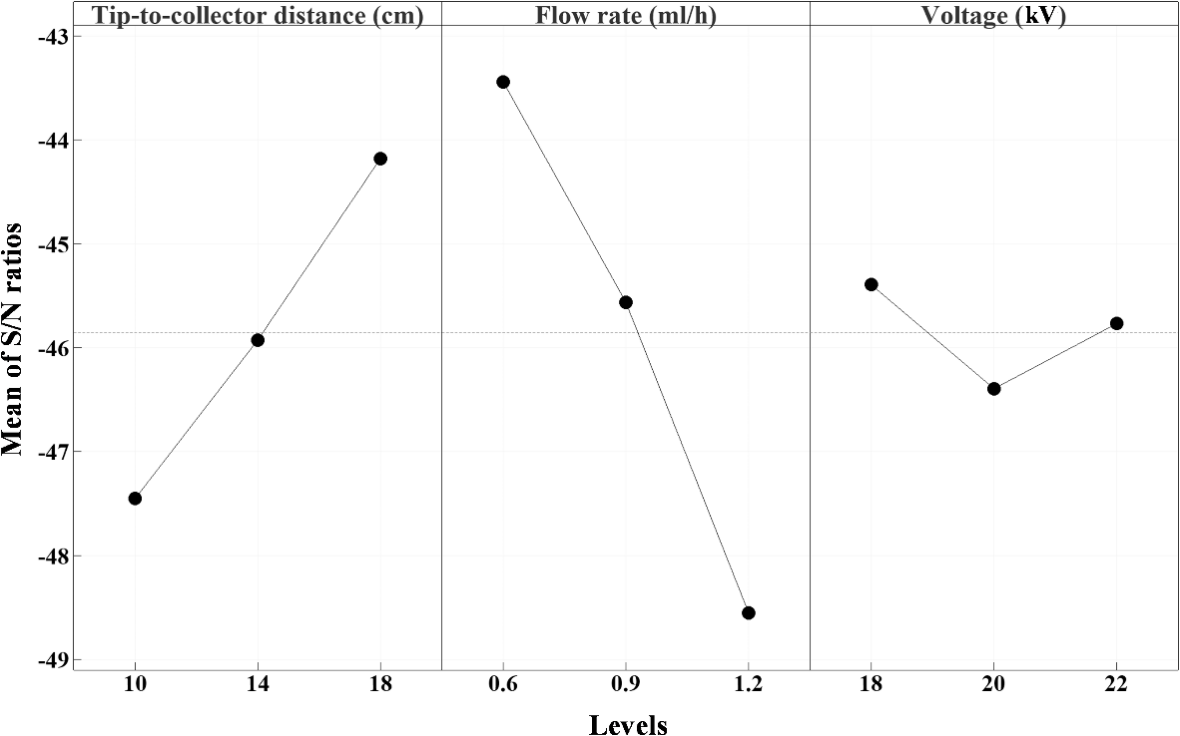
Mean signal-to-noise (S/N) ratio for different parametric design settings.

Figure 5 presents mean S/N ratio profiles for various factors at different levels, providing a comprehensive overview of their relative effectiveness. These profiles are instrumental in identifying the significance of each factor by highlighting the differences in S/N ratio values across the levels. The magnitude of variation between the highest and lowest S/N ratios for each factor indicates the degree of their impact on the outcome. As previously noted, higher S/N ratios correspond to smaller nanofiber diameters, making them a critical determinant of optimal performance. Specifically, the combination of A_18_B_0.6_C_18_ stands out as the most favorable, resulting in the smallest average diameter based on the mean S/N ratio values derived from the Taguchi method, as shown in Fig. 4. Additionally, the use of Analysis of Variance (ANOVA), a widely used statistical method, allows for a detailed evaluation of the significance of each parameter, thereby improving the precision and reliability of the analysis.

The percentage contribution (PC) can be computed as follows:

2 $$PC = \frac{{SS_{P} }}{{SS_{T} }}$$

SS_T_, or total sum of squares, pertains to the overall variance of the measurements. It encompasses the sum of squared errors (SSe) and the sum of squared deviations (SS_P_) caused by each process parameter.

Table 4 presents the results of the regression analysis, including F-values, *P* values, degrees of freedom (DF), sum of squares (SS), and mean square (MS). Notably, the *P* values for factors A and B are both less than 0.05, indicating that these factors have significant effects on the average nanofiber diameter. In contrast, factor C has a larger *P* value (> 0.05), suggesting that it has a lesser impact on the nanofiber diameter. The regression analysis was conducted with a confidence level of 95% and a significance level set at α = 0.05. The ANOVA analysis reveals a regression *P* value of 0.001 and an F-value of 32.53, demonstrating the validity of the model, as the *P* value is below 5%. Additionally, the model’s R^2^ value of 95.13% confirms its reliability. Furthermore, Table 4 presents the contribution ratio of each parameter, with the flow rate (factor B) accounting for 67% of the overall impact. This highlights the significant influence of the flow rate on the average nanofiber diameter. In contrast, factor C (voltage) has a comparatively minor effect on the average nanofiber diameter.

**Table 4:** Regression analysis.

Sources	DF	Seq SS	Contributions (%)	Adj SS	Adj MS	F-Values	*P* Values
Regression	3	30,489.7	95.13	30,489.7	10,163.2	32.53	0.001
A	1	8626.0	26.91	8626.0	8626.0	27.61	0.003
B	1	21,564.0	67.28	21,564.0	21,564.0	69.03	0.000
C	1	299.6	0.93	299.6	299.6	0.96	0.372
Error	5	1562.0	4.87	1562.0	312.4		
Total	8	32,051.7	100.00				

### Confirmation test

**Fig. 6:**
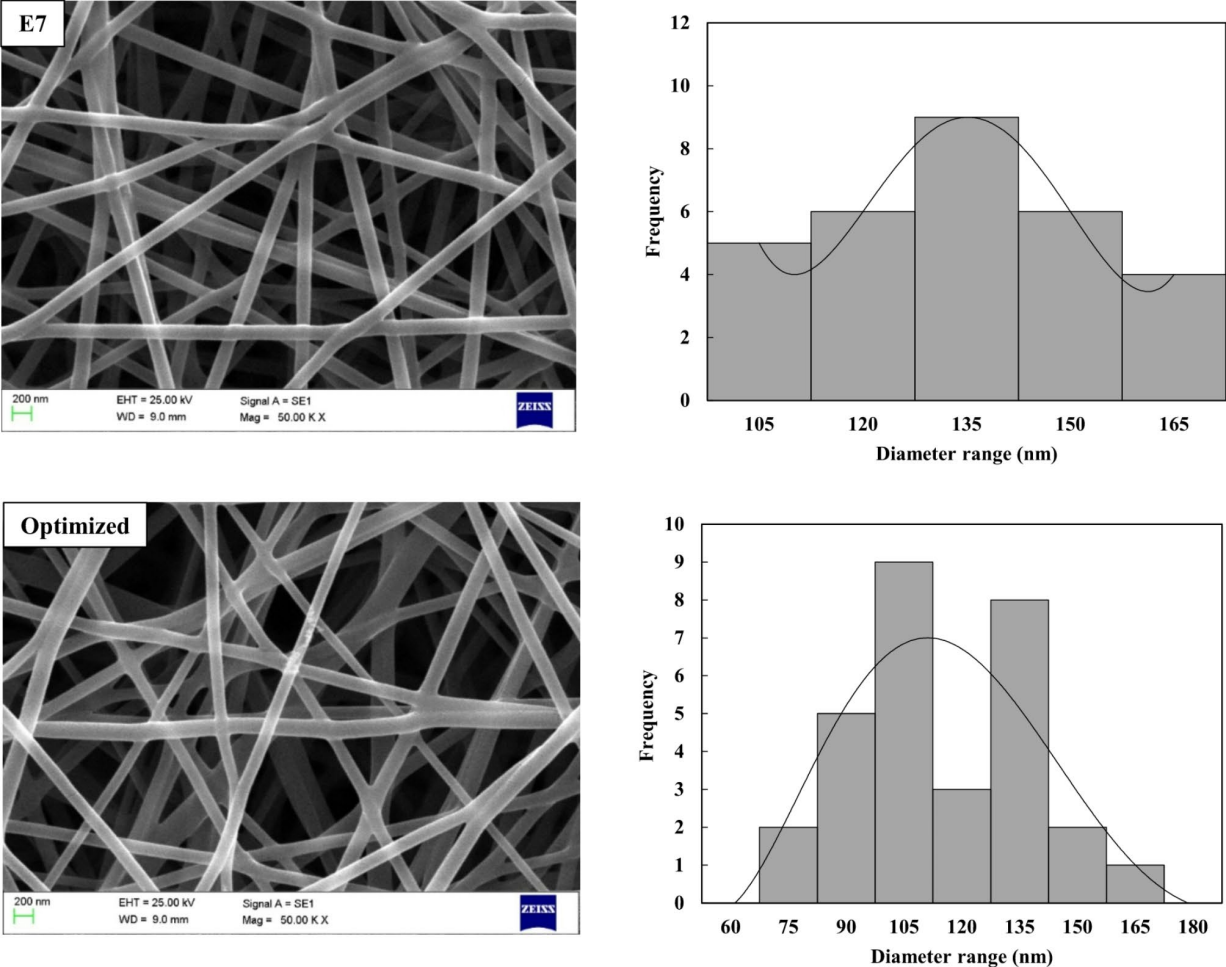
SEM images of electrospun nanofibers and their frequency distribution across diameter ranges for the initial and optimized parameter combinations.

Following the completion of the Taguchi and ANOVA analysis, a crucial step is the confirmation test to validate the optimal combination of factors and levels. This verification ensures the robustness and reliability of the results. Table 5 presents the outcomes of the confirmation test, including both the predicted and tested nanofiber diameters. Among the nine scenarios, combination E7 is initially identified as the preferred choice due to its minimal nanofiber diameter, as indicated by the S/N ratio analysis. However, it is noted that the optimal condition (E7) derived from the orthogonal array differs from the optimized combination (A_18_B_0.6_C_18_) obtained from the mean S/N ratios, as shown in Fig. 5. Therefore, a final experimental test is necessary to confirm the accuracy and consistency of the results. The data presented in the table demonstrate a remarkable consistency between the predicted and tested values, confirming the precision of the analysis. Significant improvements are observed, including a 0.8 dB increase in the S/N ratio and a notable reduction of approximately 21% in the average nanofiber diameter. Figure 6 provides a detailed comparison of the morphologies for the initial and final optimal combinations, allowing for in-depth analysis. This confirmation test is a critical step in validating the reliability and effectiveness of the Taguchi method used to optimize the design parameters, thereby reinforcing the credibility of the results.

**Table 5:** Results of confirmation test.

	Initial combination	Optimum combination	
		Predicted by Taguchi	Experiment result
Combination (factor/level)	A_18_B_0.6_C_22_	A_18_B_0.6_C_18_	A_18_B_0.6_C_18_
Average nanofiber diameter (nm)	127.6 ± 19.8	100.8 ± 28.4	106.3 ± 23.1
S/N ratio (dB)	− 42.1	− 41.3	–

### Prediction results of ANN model

**Fig. 7:**
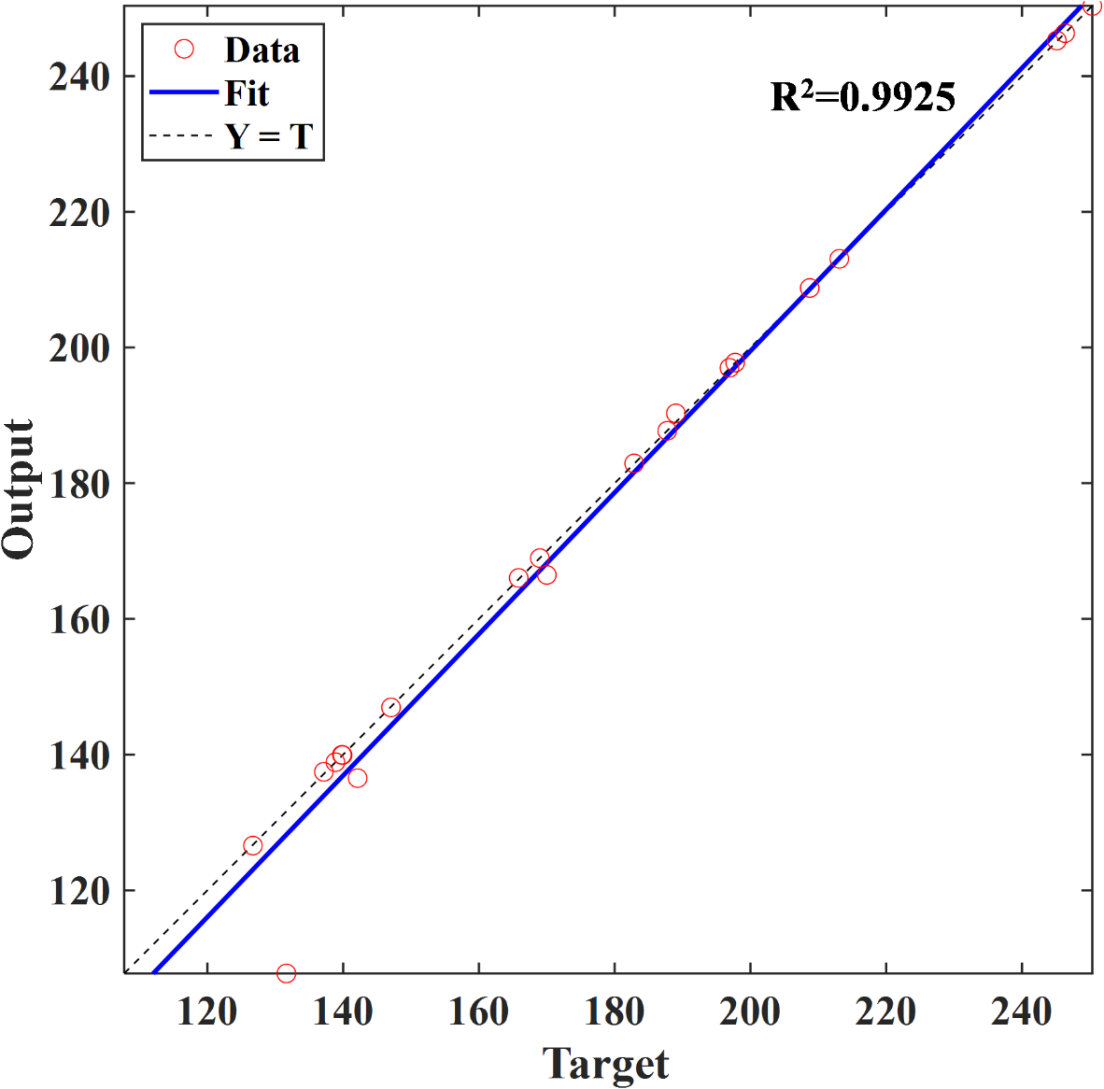
Comparison of ANN prediction and target results.

Figure 7 presents the prediction results of ANN model, which show a high level of accuracy, with an overall score of 0.9925 when compared to the target data. This strong correlation highlights the ANN model’s robust predictive capabilities, making it a reliable and efficient alternative to the computationally intensive CFD model. By providing fast and accurate predictions of nanofiber diameter, the ANN model significantly enhances the ability to implement predictive experimental outcomes.

### Regression analysis

To model the nonlinear relationship between the objective function and design input variables, a second-order polynomial regression model incorporating interaction terms is utilized. This can be formulated as:

3 $$y = a_{0} + \sum {a_{i} } x_{i} + \sum {a_{ij} } x_{i} x_{j} + \sum {a_{ii} } x_{i}^{2}$$

where $a_{0}$ represents the constant term, $a_{i}$ corresponds to the linear regression coefficient of the variable $x_{i}$, $a_{ij}$ denotes the regression coefficient for second-order interaction effect of variable $x_{i}$ with the variable $x_{j}$. $a_{ii}$ is the regression coefficient for the second-order terms of the variable $x_{i}^{2}$.

The equation of average nanofiber diameter is developed through second-order polynomial regression analysis based on 27 data points, resulting in the following Eq. (4).

4 $$\begin{aligned} {\text{Diameter}} &= - 1979.7563 - 62.4125x_{1} + 0.0000x_{2} + 268.3250x_{3} - 0.9783x_{1}^{2} \hfill \\ &\quad- 124.6605x_{2}^{2} - 10.5507x_{3}^{2} - 48.0602x_{1} x_{2} + 6.4007x_{1} x_{3} + 59.1204x_{2} x_{3} \hfill \\ \end{aligned}$$

### Mechanical properties

**Fig. 8:**
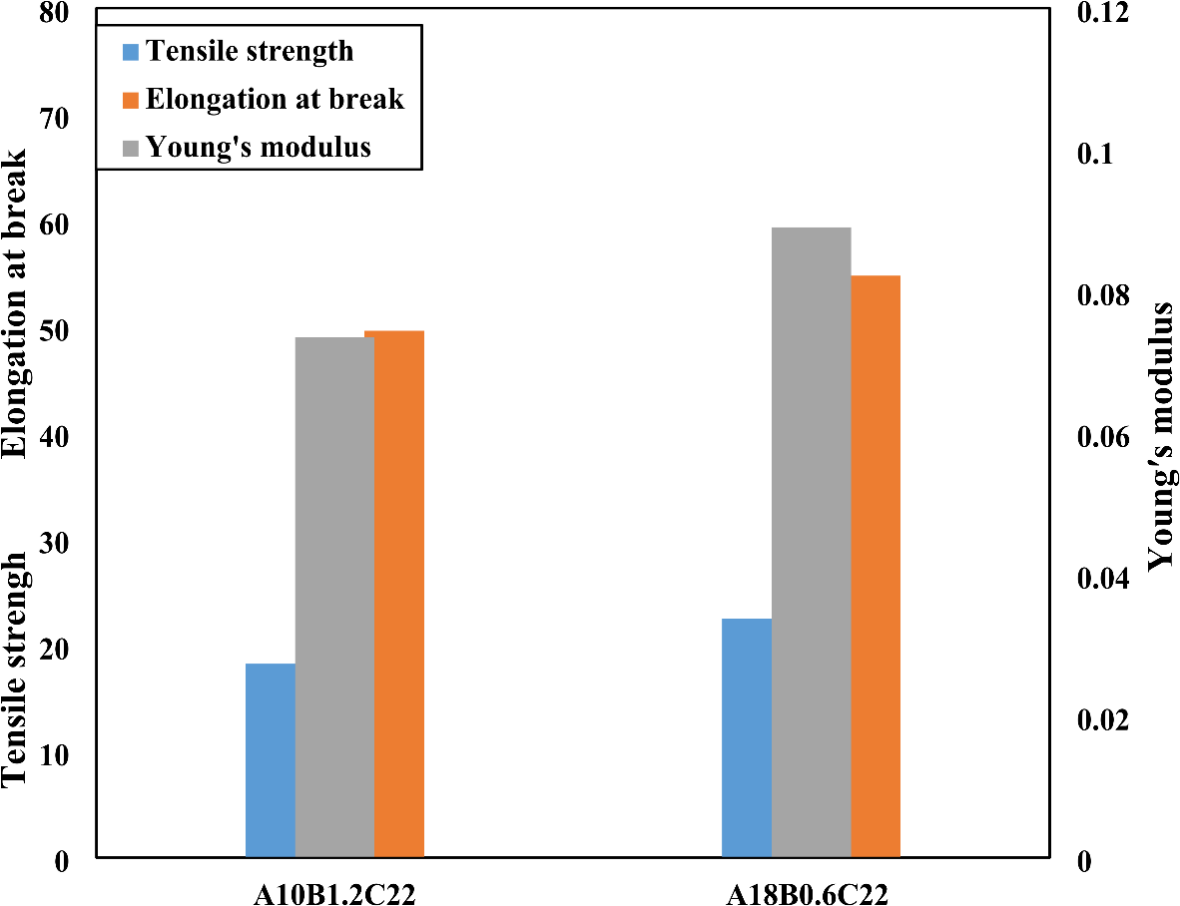
Mechanical properties of A_18_B_0.6_C_22_ and A_10_B_1.2_C_22_ nanofibers, comparing their Tensile strength, Elongation at break and Young’s modulus.

A comprehensive understanding of the mechanical properties of wound dressing materials is crucial, as these materials must exhibit specific attributes to ensure effective performance. Key properties include tensile strength, durability, flexibility, bending resistance, and elasticity. Additionally, ease of replacement is essential to prevent trauma or damage to the newly formed epithelial tissues beneath the dressing. The desired mechanical characteristics of wound dressings can be quantitatively evaluated through tensile testing, which allows for assessing the appropriate balance between flexibility and rigidity^52^. For example, Fig. 8 illustrates the tensile strength, elongation at break, and Young’s modulus of polyvinyl alcohol (PVA) and polylactic acid (PLA) nanofibers blended in a 50:50 ratio, under two different parameter conditions: A_18_B_0.6_C_22_ and A_10_B_1.2_C_22_. The results demonstrate that the combination of PVA and PLA polymers creates an overlap of their respective mechanical properties, resulting in nanofibers with enhanced overall performance.

Notably, the nanofibers produced under the A_18_B_0.6_C_22_ conditions, with an average diameter of 127.6 ± 19.8 nm, exhibited superior mechanical properties when compared to those produced under the A_10_B_1.2_C_22_ conditions, which had a larger average diameter of 307 nm. This difference in diameter suggests that the finer fibers may improve both the overall mechanical strength and flexibility, making them particularly suitable for wound dressing applications. These findings highlight the importance of optimizing both the composition and fabrication parameters to achieve the ideal mechanical properties for effective wound care solutions.

### Contact angle of water

**Fig. 9:**
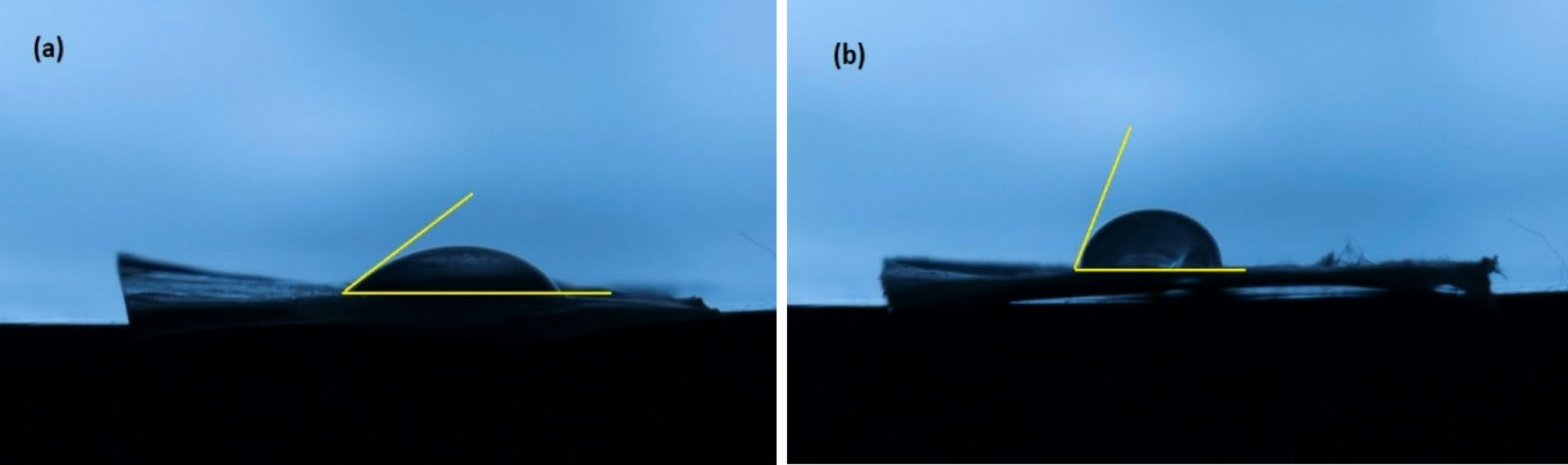
Water contact angle measurements of electrospun nanofibers: (**a**) A_18_B_0.6_C_22_ and (**b**) A_10_B_1.2_C_22_, illustrating their surface hydrophilicity.

The measurement of the water contact angle is the most widely used method for evaluating the wettability of materials, providing valuable insights into their surface characteristics. A solid surface is classified as hydrophobic if the contact angle exceeds 90°, whereas surfaces with contact angles below this threshold are considered hydrophilic. Hydrophilicity is a crucial factor influencing the cytocompatibility of biomaterials, as it significantly affects cell adhesion and growth. Specifically, cells tend to preferentially adhere to hydrophilic surfaces, making wettability an essential consideration in the design of biomaterials for medical applications^53^. In this study, water contact angles were systematically measured to evaluate the hydrophilicity of two types of scaffolds: A_18_B_0.6_C_22_ and A_10_B_1.2_C_22_. The results, illustrated in Fig. 9, reveal notable differences in hydrophilicity between the two scaffolds. The A_18_B_0.6_C_22_ scaffold, characterized by an average diameter of 127.6 ± 19.8 nm, exhibits a water contact angle of 37°, signifying a high degree of hydrophilicity. In contrast, the A_10_B_1.2_C_22_ scaffold, with a larger average diameter of 307.6 ± 57.5 nm, displays a contact angle of 72°, indicating reduced hydrophilicity.

These findings suggest that the finer A_18_B_0.6_C_22_ scaffold may enhance cell adhesion and proliferation due to its favorable wettability characteristics. The variations in contact angle measurements emphasize the significance of scaffold morphology and surface properties in influencing biological interactions. This underscores the importance of optimizing these parameters to enhance the performance of biomaterials in tissue engineering and regenerative medicine applications. Overall, understanding the relationship between surface wettability and cellular behavior is crucial for developing effective biomaterial scaffolds that facilitate successful tissue integration and promote healing.

## Conclusions

This study optimized the electrospinning of PVA/PLA nanofibers for wound dressing applications, using a mutual solvent. By varying key parameters—needle tip-to-collector distance, flow rate, and applied voltage through an L9 orthogonal array and the Taguchi method—the nanofiber diameter was reduced. An ANN-based surrogate model is developed using the dataset generated through DoE to efficiently predict experimental outcomes, streamlining the identification of optimal design configurations. ANOVA analysis showed that flow rate had the most significant impact on diameter, while applied voltage had minimal effect. The optimal configuration (A_18_B_0.6_C_18_) produced nanofibers with a mean diameter of 103.4 nm. These smaller nanofibers offer advantages such as increased surface area for better cellular interactions, moisture management, and drug loading, as well as enhanced mechanical properties like tensile strength and flexibility. The A_18_B_0.6_C_22_ scaffold showed superior hydrophilicity, improving cell adhesion and proliferation. These optimized PVA/PLA nanofibers demonstrate promise for advanced wound care, with improved mechanical and wettability properties that support cellular processes and wound secretion absorption, making them suitable for regenerative medicine and next-generation medical devices.

These optimized PVA/PLA nanofibers exhibit significant potential for advanced wound care applications, offering enhanced mechanical integrity, wettability, and biofunctionality that support cellular processes and efficient wound exudate management. While this study establishes a robust foundation, future research will focus on refining the ANN model by incorporating additional critical parameters, including ambient environmental conditions, solvent system interactions, and polymer composition variability. Expanding the model’s scope will not only enhance predictive accuracy but also enable a more comprehensive understanding of the complex, nonlinear relationships governing fiber morphology. This progressive approach will facilitate the development of more versatile electrospun scaffolds with precisely tailored properties, ultimately advancing their applicability in regenerative medicine, controlled drug delivery systems, and next-generation biomedical devices.

## Data Availability

The datasets used and/or analysed during the current study available from the corresponding author on reasonable request.
